# Ecosystem Composition Controls the Fate of Rare Earth Elements during Incipient Soil Genesis

**DOI:** 10.1038/srep43208

**Published:** 2017-02-23

**Authors:** Dragos G. Zaharescu, Carmen I. Burghelea, Katerina Dontsova, Jennifer K. Presler, Raina M. Maier, Travis Huxman, Kenneth J. Domanik, Edward A. Hunt, Mary K. Amistadi, Emily E. Gaddis, Maria A. Palacios-Menendez, Maria O. Vaquera-Ibarra, Jon Chorover

**Affiliations:** 1 Biosphere 2, The University of Arizona, Tucson, AZ, USA; 2 Department of Soil, Water & Environmental Science, The University of Arizona, Tucson, AZ, USA; 3 School of Biological Sciences, University of California, Irvine, CA, USA; 4 Lunar and Planetary Laboratory, The University of Arizona, Tucson, AZ, USA; 5 Arizona Laboratory for Emerging Contaminants, The University of Arizona, Tucson, AZ, USA; 6 Williams College, Williamstown, MA, USA; 7 The University of Caribe, Cancún, México; 8 University of the Americas Puebla, Puebla, México

## Abstract

The rare earth elements (REE) are increasingly important in a variety of science and economic fields, including (bio)geosciences, paleoecology, astrobiology, and mining. However, REE distribution in early rock-microbe-plant systems has remained elusive. We tested the hypothesis that REE mass-partitioning during incipient weathering of basalt, rhyolite, granite and schist depends on the activity of microbes, vascular plants (Buffalo grass), and arbuscular mycorrhiza. Pore-water element abundances revealed a rapid transition from abiotic to biotic signatures of weathering, the latter associated with smaller aqueous loss and larger plant uptake. Abiotic dissolution was 39% of total denudation in plant-microbes-mycorrhiza treatment. Microbes incremented denudation, particularly in rhyolite, and this resulted in decreased bioavailable solid pools in this rock. Total mobilization (aqueous + uptake) was ten times greater in planted compared to abiotic treatments, REE masses in plant generally exceeding those in water. Larger plants increased bioavailable solid pools, consistent with enhanced soil genesis. Mycorrhiza generally had a positive effect on total mobilization. The main mechanism behind incipient REE weathering was carbonation enhanced by biotic respiration, the denudation patterns being largely dictated by mineralogy. A consistent biotic signature was observed in La:phosphate and mobilization: solid pool ratios, and in the pattern of denudation and uptake.

The transformation of upper crust to soil supports Earth’s terrestrial life. Soil genesis and evolution have been under scientific scrutiny for over a century. With recent advances in analytical chemistry, molecular and evolutionary biology, hydrology, ecology and remote sensing we are only now beginning to understand how different components of geosphere, hydrosphere, atmosphere, and biosphere work together at different scales to shape Earth’s surface and transform parent rock into soil that sustains ecosystems^1^,^2^,^3^. Interactions among chemical, biological, physical and geological processes are central to the critical zone conceptual framework, which seeks to advance knowledge on the coupled processes driving the biogeochemical evolution of the shallow and porous crustal environment supporting life^4^.

The igneous rock, Earth’s nutrient store, exhibits its largest thermodynamic disequilibrium and becomes highly susceptible to weathering when exposed to the oxic and aqueous environment of the surface. The earliest stages of mineral weathering are among the most reactive, and interactions with first microbial and plant communities initiate the flow of energy and nutrients which feed the major biogeochemical cycles. Rapid transformation of mineral surfaces often accompanies the early stages of weathering, and it is intensified under colonization by microbiota and plant roots. The transformation includes both physical weathering, such as mechanical micro-fracturing driven by differential mineral expansion (e.g. Fe oxidation in biotite and pyrite, mineral hydration, temperature) and chemical reactions (dissolution of primary minerals and precipitation of secondary minerals). With time, rates decrease due to a decrease in surface area of the most reactive primary minerals, solution phase saturation with respect to the constituent minerals, and physical occlusion of primary minerals by secondary mineral precipitates^5^. All of these can control the rates of chemical weathering, which span over six order of magnitude from the lowest rates observed in natural field settings (with aged surfaces and close-to-equilibrium solutions) to the highest experimental rates (fresh rock with reactive fine particles and generally far-from-equilibrium conditions)^5^. Because of this, higher physical denudation (or removal of weathered rock) can result in increased chemical weathering rates^5^.

In natural weathering environments elements display distinct geochemical behavior in accordance with different classes of reactivity, e. g., conservative elements present in recalcitrant solids (Zr and Ti), low mobility elements preferentially incorporated into secondary-phase weathering products (Fe, Al, Si), cations liberated into pore fluids during silicate weathering and adsorbed to clay mineral surfaces (K, Mg, Na and Sr), as well as biolimiting elements (P) and lanthanides^6^. In the natural soil-forming system studying these early interactions has classically been achieved using nutrients that are part of plant and microorganism metabolic budgets. These studies have shown, for example, preferential dissolution and loss of major elements such as Fe, Na and Ca from micaceous minerals and plagioclase^7^,^8^, microbe and plant elemental uptake, and incorporation into newly formed minerals^9^,^10^. Resulting biological signals of these elements in the complex porous geomedia can, however, be masked by their high and diverse reactivity, and competing chemical processes. The use of less mobile elements as indicators of biological weathering is potentially more powerful because of their simpler cycle and the comparatively stronger selective force organisms would need to affect their stoichiometry.

Rare earth elements (REE) are the lanthanide series (atomic numbers [Z] 57 [lanthanum] to 71 [lutetium]) with yttrium (Z = 39) often included because its outer electron shell structure and ionic radius are nearly identical to holmium^11^. REE exhibit generally similar but highly dispersed environmental distributions (rarely forming mineral deposits). Coherent trends in their aqueous reactivity derive from the similar stable arrangement of outer electron configuration (5*sp*) across the series, superimposed with ionic radius variation resulting from gradual filling of the inner 4f electron shell. The lower polarization of empty (La, Y), half-filled (Gd) and filled (Lu) 4f electron shells, as well as variable redox states of Ce and Eu can lead to radius-independent partitioning behavior among elements^12^.

The series exhibits a consistent decrease in ionic radius with increasing atomic mass (lanthanide contraction effect). This effect, along with slight differences in ionic potential and unpaired 4f electrons, can induce variability in chemical behavior among REE^13^,^14^. Some differences in individual REE partitioning among rock, solution, secondary minerals, and biomass in low temperature natural terrestrial systems is therefore possible, particularly at circumneutral pH^15^, due to differential dissolution of minerals^16^, surface adsorption reactions of dissolved REE^17^, co-precipitation in secondary minerals (e.g., silicate clays and Mn-, Al-, Fe-(oxy)hydroxides^18^), competition with major ions on organic binding sites^19^ and soluble complex formation with a variety of ligands, including carbonates and dissolved organic matter^16^. Likewise, smoothed curves along REE series (tetrad effect) have been described in shale-normalized REE series, indicating their involvement in ionic radius/charge independent processes^20^. The tetrad effect is presumably related to the increased stability at quarter, half, three-quarter, and complete filling of the 4f electron shell^21^. Systematic variation in REE reactivity across the series, uncommon to other elements, has stimulated their use as tracers of a variety of geochemical processes, from mantle and crustal to cosmogenic evolution, ore genesis, sedimentary petrology^22^, water-rock interactions^23^, and critical zone evolution^24^.

We postulate that since REE form strong complexes with bio-ligands^24^,^25^, ecosystem components (microbes and plants) can affect the behavior of REE during weathering, e.g., by selective ligand-complexation of dissolved REE ions. REE cycles in the biosphere are still poorly understood, with conflicting evidence and opinions regarding biological effects on their cycles and their role in biological systems. In regard to the latter, REE were shown to stimulate plant biomass production^13^, cause disruption of physiological functions, e.g. floral development and photosynthesis by replacing major functional metals such as Ca in a nutrient-deprived environment^26^,^27^, and undergo biosorption by microorganisms^28^. Moreover, arbuscular mycorrhiza, the most widespread type of soil fungal-plant symbiosis associated with 74% of flowering plant species^29^, can both, reduce and increase the extraction of REE ions from mine tailings^30^. Adding to this debate is a report of lanthanides being essential for some acidophilic methanotrophic microbes by providing superior catalytic properties to dehydrogenase proteins^31^. However, biological effects on incipient REE cycles in natural conditions are practically unknown.

While distribution patterns along the REE series in solid and water have the potential to encode signatures of processes leading to mineral dissolution and secondary mineral formation^32^, much less is known about the role of this class of trace elements in the incipient biological transformation of Earth’s crust. Hypothetically, due to their similar geochemical behavior in host minerals and their reactivity with diverse biological ligands, REE could be suitable long-term tracers of a wide range of biological rock alterations. Knowledge of such behavior may provide signatures of life’s presence and the extent of its influence on bedrock on early and modern Earth, and potentially on other planetary bodies.

Here we present results from a highly controlled experiment designed to quantify the extent to which variation in the nature of incipient weathering by different ecosystem components (microbes, vascular plant and arbuscular mycorrhiza) results in the mobilization and redistribution of REE during silicate rock (igneous and metamorphic) weathering. We hypothesized that rock type and the nature of rock-colonizing biotic communities would affect REE partitioning and that differential and reproducible inter-elemental patterns would, therefore, be observed in pore waters, biological tissue, and solid-phase extractable pools. Specifically, we tested whether: (a) rock substrate controls both chemical and biological REE denudation in pore water; (b) there is a distinct signal of biotic presence in pore water REE content; (c) there is selective uptake and distribution of REE in plants, (d) plant REE uptake is affected by the presence of arbuscular mycorrhizal fungi; and (e) there is a biotic influence on the soluble and poorly-crystalline mineral pools.

## Results and Discussion

### Rock substrates and REE sources

**Figure 1:**
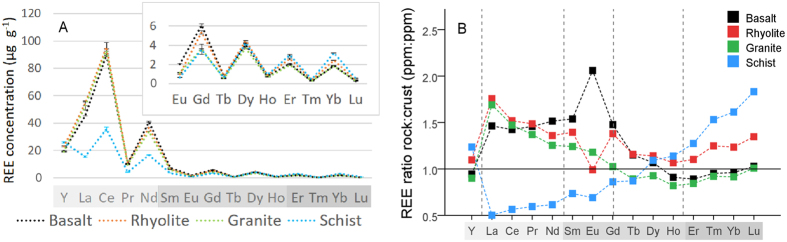
REE abundances in the original rock. (**A**) REE concentrations in bulk rocks. (**B**) The concentration of REE in the initial rock substrates normalized to Earth’s upper continental crust average^33^. Values above unity represent enrichment, while those below, represent depletion. Tetrad groups (except Y) are separated by vertical dashed lines. Elements are arranged by increasing atomic number, with those defined as low (L), medium (M) and heavy (H) atomic mass displayed on X axis with light, medium and dark gray background, respectively.

Initial concentrations of various REE in the four rocks were similar except for schist, which had less light (L)-REE and more heavy (H)-REE than the other rocks (Fig. 1A). Schist was also depleted in L-REE relative to upper continental crust while basalt, rhyolite, and granite were enriched (Fig. 1B). A weak M-class tetrad effect^20^ was observed in granite (Fig. 1B). Europium exhibited a positive anomaly in basalt and a negative one in rhyolite. In basalt, microprobe analysis identified Ce-Nd-La-Pr oxides as mineral hosts for L-REE, and the glass matrix as the source of heavier REE (Fig. s3). Principal hosts in rhyolite included ilmenite (rich in La, Gd, and Yb) and apatite (rich in Y). In granite, sphene, apatite (both rich in L-REE) and K-feldspars were predominant hosts, whereas in schist, zircon (rich in Yb), allanite (rich in La) and xenotime (rich in Y) were prevalent. These differences in bedrock chemistry and mineralogy are expected to influence the stoichiometry of REE release during weathering.

### REE denudation in solution from time 0 to 20 months

**Figure 2:**
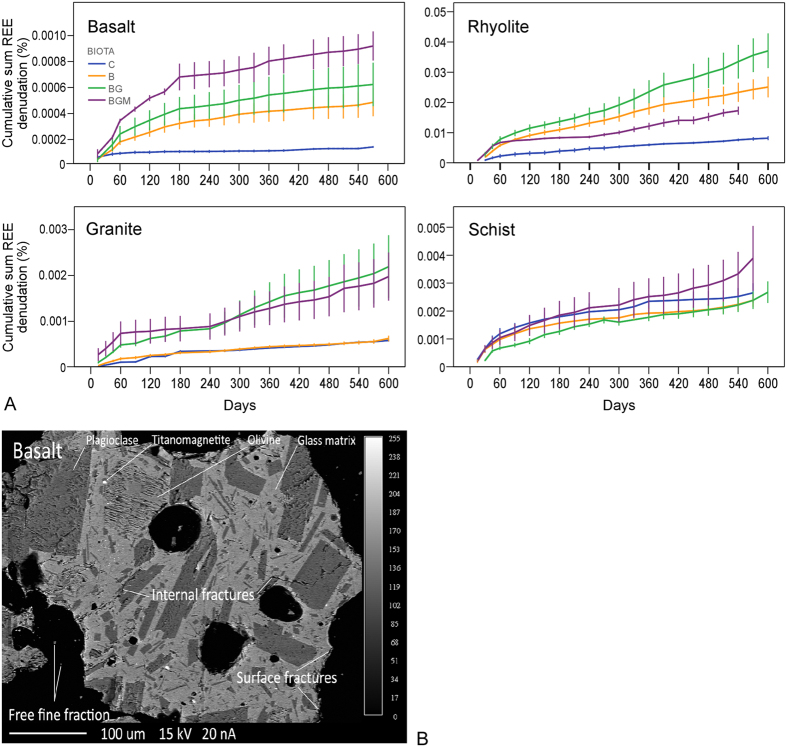
REE preferential denudation over time under abiotic and biotic influence and physical changes in the rock. (**A**) Cumulative denudation per column for a sum of all REE relative to their rock content. Values are means of three replicate columns and error bars represent one standard error of the mean. **C**, control; **B**, bacteria; BG, bacteria-grass; BGM, bacteria-grass-mycorrhiza. (**B**) An example of micro-fracture development during incipient weathering of basalt, as shown by electron microprobe imaging of a grain extracted at the end of the experiment from abiotic control.

When exposed to water under condition of room temperature, atmospheric gas concentrations and humidity, all rocks exhibited rapid REE release to aqueous solution (i.e., chemical denudation), which slowed down after the initial two months of the experiment (Fig. 2A). Microbes and plants induced greater denudation than the abiotic control in basalt and rhyolite. The presence of arbuscular mycorrhiza enhanced denudation in comparison to the plant-microbe condition alone in basalt and schist, but reduced it in rhyolite, probably related to REE retention in pore space. In granite, bacteria alone had no effect; only planted treatments significantly increased denudation *vs* control. Also in schist, microbes and microbes-grass treatments exhibited lower chemical denudation than control. A significant divergence between treatments, where present, started to develop in pore water within 4–6 weeks of seeding and/or inoculation (Fig. 2A).

The observed pattern of denudation is consistent with a two-phase process: an initial highly reactive phase, when readily available sites on fresh mineral surfaces release REE to pore waters through mostly abiotic water-rock interactions, e.g. hydrolysis or carbonation; followed by a phase with slower increase in denudation but greater biological influence. Previous observations of major element (Si, Ca and K) leaching from materials of comparable particle size and mineralogy have shown high initial removal in biota and biota-free substrates followed by a steady-state phase^34^. This is consistent with our measured changes in root growth; in the first 120 days, roots accumulated 0.12 ± 0.06 g of biomass per column, or 1198 ± 327 cm total root length, while at the end of the experiment (~600 days) they measured 0.14 ± 0.04 g and 2728 ± 2577 cm, indicating that majority of plant growth occurred during the first four months, possibly limited by the weathering rate.

Thermodynamic modelling of solution phase saturation with respect to REE-bearing solid phases in collected pore water using Visual Minteq 3.1 by KTH (J. P. Gustafsson; https://vminteq.lwr.kth.se/) showed that pore waters were consistently undersaturated with respect to REE oxides throughout the experiment. With the input of nano-pure water, monthly flushing of pore space, relatively large grain size of the substrate, and actively growing biota in the nutrient-limited environment, it is unlikely that approach to geochemical equilibrium was the cause of decreased rates of weathering. Alternative possibilities that can explain decrease in dissolution rates, such as formation of passivating layers (both organic and inorganic) around the mineral surfaces, are possible, but were not detected during such early stages of soil development (Fig. 2B). Capillary micro-fractures on grain surfaces, as well as nano- and microparticles (Figs 2B and s3) may have played an important role in the initial highly reactive period by increasing surface exposed to weathering. With time, surface roughness should decrease and the smallest primary mineral particles (with highest surface area) should be preferentially removed by dissolution.

Total REE mass removed in solution differed by rock (Table s2). Rhyolite showed the highest REE denudation followed by schist, granite, and basalt. Biological treatment effects observed for REE were also observed for other water parameters, including electrical conductivity (EC). For example, basalt, which showed the greatest REE denudation for combined planted treatments relative to control also had higher EC in planted treatments, indicating greater weathering overall. Other potential indicators of weathering, such as solution pH and total dissolved organic carbon were less sensitive to the biological effect (Table s2).

**Figure 3:**
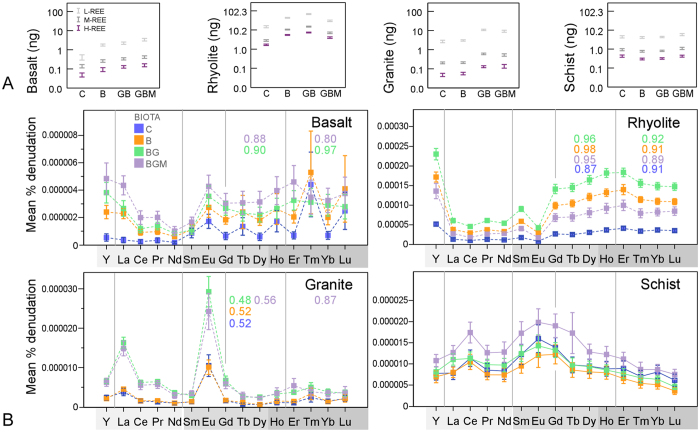
Abiotic and biotic influence on REE variability in pore-water. (**A**) Total denudation of L-, M- and H-REE in pore water collected over the 20-month experiment for following treatments: C, control; B, bacteria; BG, bacteria-grass; BGM, bacteria-grass-mycorrhiza. (**B**) Rock normalized sample means of L-, M- and H-REE in pore-water (g in water : g in rock, expressed as % per column) as a function of rock and ecosystem composition. Error bars represent one standard error (SE) of the mean. Tetrad groups (Y excluded) are separated by vertical lines. Numbers inset represent tetrad magnitude values, with M- and W-type curve values above and below 1, respectively. Values close to unity (no tetrad effect) are not displayed. Number colors correspond to treatment colors.

Trends observed for the sum of REE (Fig. 2A) were also observed for groups of REE (Fig. 3A) and for individual elements (Fig. 3B). Among groups, L-REE experienced greatest denudation followed by M-REE and H-REE (Fig. 3A), consistent with their rock abundances (Fig. 1A). Rock-normalized REE concentrations in solution revealed similar effects as in time-lapse analysis, with the notable exception of schist where the biotic effect of vascular plants (BG) over microbes (B) and mycorrhiza (BGM) over BG was clearer, particularly for L-REE (Fig. 3B). These differences between biological treatments with substrate imply an important rock-dependent biological effect on REE released from rock during weathering.

A major depression occurred in the L-REE segment in basalt and rhyolite, which was enhanced by biological treatments (Fig. 3B). Since both rocks were relatively rich in these elements (Fig. 1), the depressions could be caused by comparatively slower dissolution kinetics of host minerals and higher uptake. For instance, in basalt, previous studies showed that amorphous glass, the identified source of H-REE in our experiment (Section 2.1 and Fig. s3), is among the first constituents to dissolve^1^,^34^. This would release higher amounts of H-REE in solution, producing a depression in L-REE. In granite, preferential dissolution of apatite^35^, targeted by plant and mycorrhiza due to its rich P content^36^, is likely responsible for relatively high L-REE values observed in water collected from this rock (Fig. 3B).

Europium and cerium are known to exhibit anomalies due to their variable oxidation states (Eu separates during precipitation from magma, and Ce during low-temperature water-mineral interaction). During dissolution, Eu anomalies were recorded for pore waters effluent from the basalt, granite (even though no Eu anomaly was present in parent granite; Fig. 1B) and rhyolite, and a Ce anomaly was observed for the schist treatment (Fig. 3B). An Eu anomaly is common during aqueous weathering of felsic rock, particularly when organic acids are present^37^, and can be attributed to Eu preferential release from feldspars and apatite, where Eu^2+^ can substitute for Ca^2+ ^13. Cerium - the only lanthanide that undergoes oxidation from more soluble +3 to less soluble +4 valence state in low temperature aqueous environments^38^, correlated with Fe (*r* = 0.45), Ti (*r* = 0.42) and P (*r* = 0.38); *p* < 0.05 in schist, consistent with their leaching from accessory minerals allanite and xenotime. The results also showed plant-enhanced peaks of La in granite and of Tm in basalt. The effect on La can stem from its electronic structure (no unpaired 4f electrons associated with largest ionic radius) which imprints the lowest ligand complexation potential among REE^25^.

Except for schist, a subtle W-class tetrad effect (concave) overlapped the broader patterns in water (Fig. 3B). The most conspicuous are the third and fourth tetrads in basalt, clearly developed under plant and mycorrhiza. W-tetrad depressions, presumably produced due to decreasing strength of REE ion-ligand complexes during mineral-water reactions^39^ have been reported in water-rock interactions and mineralizing hydrothermal fluids^20^,^40^ and have been described in relation to a complementary M-class (convex curve) pattern^20^,^40^ developed during the same partitioning event. Our results from erupted igneous rock indicate that ecosystem activity can lead to W-class tetrads during mineral dissolution.

Surprisingly, the factors most strongly (*p* < 0.05) explaining REE dissolution mechanisms included inorganic carbon forms and host mineral major ions (Table s3). While studies in forested environments have found a strong dissolved and colloidal organic carbon control on REE mobilization^24^, its low influence in this experiment (Table s3) means that during incipient soil genesis, inorganic complexation (driven by H_2_O and dissolved CO_2_) dominates the mobilization of REE and potentially other metals, this being enhanced by the respiration of organisms (which leads to elevated partial pressure of CO_2_ in equilibrium with pore waters). This is also supported by a significant increment (*p* < 0.05) in inorganic carbon in water collected from planted treatments. Correlative findings were reported in aquifer studies, where due to less abundant organic ligands in solution, REE complexation seemed dictated by carbonate species or, in their absence, by sorption to Fe oxy-hydroxide surfaces^16^,^41^. It is, however, expected that the influence of organic carbon increases with time, as the ecosystem accumulates more below ground-biomass and organic matter.

### Biological Signature Index (BSI)

**Figure 4:**
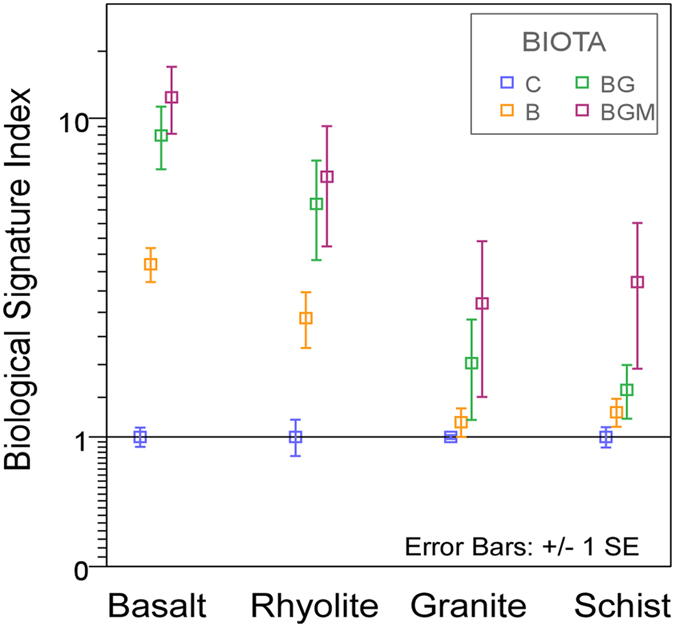
Biotic signature of REE weathering. Lanthanum/phosphate (abiotic-normalized concentrations) in pore water samples collected from the four rock types, averaged across the two-year experiment. The unit-less index was calculated by Equation s1. Treatments: C, control; B, bacteria; BG, grass-bacteria and BGM, grass-bacteria-mycorrhiza. Index values, extending from 1–100 were normalized to the abiotic control of each rock type.

The interaction between organisms and nutrient-providing substrates modifies the mobilized element balances by preferential dissolution and uptake, and this may be captured as a fingerprint in the environment. Results of abiotic control-normalized La:phosphate index (Equation s1) showed surprisingly consistent behavior among the studied substrates and biota, with abiotic values in the lowest range followed, in order of ecosystem complexity, by microbial alone, microbial and plant, and microbial, plant and mycorrhizal (Fig. 4). The strongest signals were in basalt and rhyolite, followed by schist and granite. Phosphates are major REE-bearing minerals, and P is also the life-limiting macronutrient sourced from rock^42^, as it cannot be fixed from the atmosphere. The results are consistent with preferential loss of La- one of the most reactive REE in the cationic series, from its phosphate matrix by complexation with negatively charged biological ligands. This, together with a higher water-normalized La than P found in plants, suggests a more soluble La *vs* P in the presence of biota, hence the recorded signal.

### REE uptake and distribution in plant tissue as affected by rock and mycorrhiza

**Figure 5:**
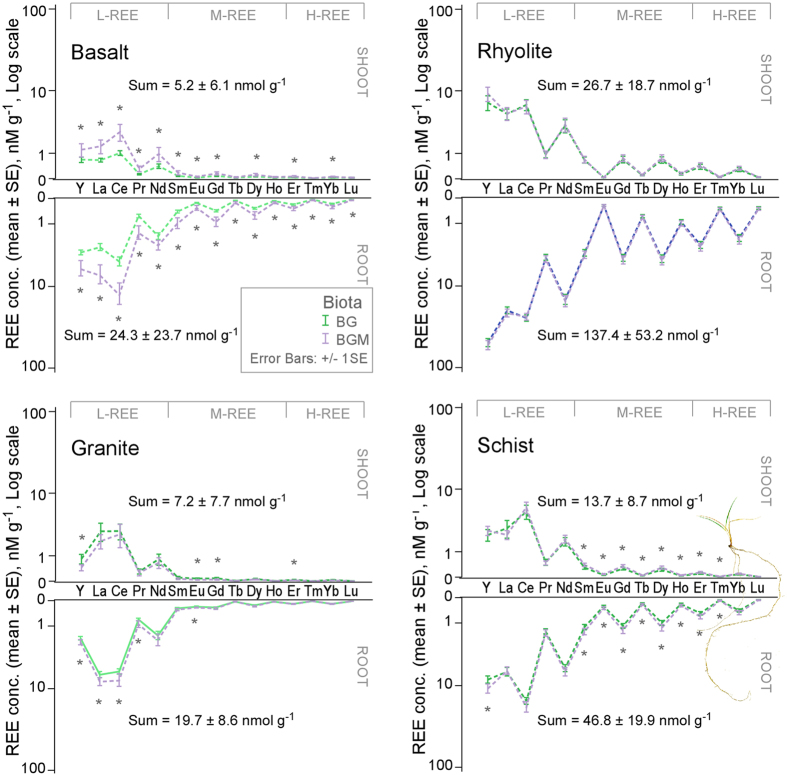
Mycorrhiza effect on REE concentration in plant organs. REE molar concentrations (nmol g^-1^) in below (root) and above (shoot) ground biomass of mycorrhiza inoculated and non-inoculated grass growing on the 4 substrates. Significant differences at ± 1SE are marked by (*).

REE concentrations in biomass were order of magnitude higher in below-ground as compared to above-ground biomass for most rocks (Fig. 5), and they were consistent with values described for natural environments^43^. Plants grown in rhyolite accumulated the highest concentrations, followed by schist, basalt, and granite. Shoot abundances generally mirrored their root counterparts.

The ratio of shoot to root concentrations followed the order (REE mean ± SE): granite (0.95 ± 0.41) ≥ basalt (0.47 ± 0.24) > schist (0.21 ± 0.01) ≥ rhyolite (0.18 ± 0.03). Granite’s high above-ground REE transfer is most likely due to its low root uptake (Fig. 5). The lower but correlative above-ground abundances are evidence of uptake/transfer controlling mechanisms that work similarly for all rare earth ions. A ring-like cell wall modification in root endodermis (Casparian strip), which protects the xylem from passive (pericellular) diffusion of solutes during water uptake, is thought to restrict the direct transfer of REE to xylem^44^,^45^ by redirecting the water flux through the selectively permeable plasma membrane of endoderm. This mechanism keeps ions in the more active aerial organs in physiologically-relevant balance regardless of their environmental abundances, thus preventing potentially toxic levels. The cell wall of root cortex (hosting negative charges of carboxyls and hydroxyls) is the most likely structure to retain REE entering the root in our experiment and it has been shown to accumulate REE ions^46^. From our results, the membrane also appears to be more permeable at lower available concentrations, e.g. granite’s higher transfer factor, which implies increased restriction at potentially toxic levels, a mechanism that may be common to other heavy elements.

Mycorrhiza increased root uptake in basalt (most REE) and schist (M- and H-REE), and REE transfer to shoot tissue (Fig. 5). Basalt also had the highest mycorrhiza infection rate, 67 ± 18% (Table s2). In rhyolite, no mycorrhiza effect on REE concentrations in biomass was found despite a 52 ± 30% infection rate and lower water REE abundances (Fig. 3), possibly due to larger biomass in this treatment (*p* < 0.2; Figure s4); hence larger total uptake than non-mycorrhiza. Plants in granite failed to develop infection (Table s2). The few studies addressing the effect of arbuscular mycorrhiza on plant REE uptake are generally in the context of crop phytotoxicity, and they showed either a decrease^47^ or increase^48^ in L-REE uptake. Our study on natural REE abundances indicates that fungal symbiosis can stimulate phyto-uptake and transfer.

**Figure 6:**
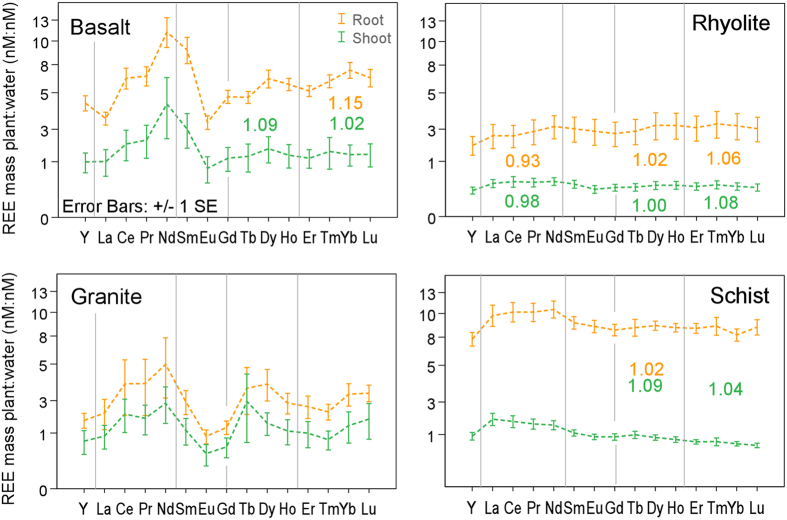
Preferential REE uptake from pore water and transfer in plant. Distribution of water normalized REE (total nmoles per column: total nmoles per column, plant : water) in above (shoot) and below (root) ground biomass. Tetrad classes are delimited by vertical lines. Numbers inset represent tetrad magnitude values (numbers range from 1-no tetrad to >1 for M-type and <1 for W-type curves), with color representing the plant organs.

Water-normalized element abundances in plant tissue (preferential uptake) showed a high uptake efficiency (molar ratio root : water of >1), and supported the concentration findings, lighter elements being favored (Fig. 6). This is similar to other vascular plants^43^,^44^, and can be ascribed to preferential uptake of the more soluble forms, or free ions^49^, owing to lower stability constants of L-REE with inorganic and organic ligands^50^. The shape of the curves generally mirrored their aquatic counterparts (Fig. 3), with a subtle M-type (convex) tetrad pattern among H-REE. This indicates that plants were key for the formation of patterns in the water. The tetrads, also found in wheat grown hydroponically with phosphate in the absence of organic ligands^51^, can be produced by the variable adsorption and/or co-precipitation of REE with phosphates and oxyhydroxides on root cell walls^46^,^52^. This is supported by the REE relationship with P and a limited number of minor (Fe, Sr, Mn, Cu, Ti, Zn, Cr, Al, Ti, and Zn) and major (Na, K, Si and Ca) elements in roots (Table s4). These root processes and patterns also appear to determine REE partitioning in aerial parts (Fig. 6). An absence of Eu and Ce anomalies in the plant, means that their dissolution mechanisms have not affected the uptake.

### REE in easily available pools in weathering rock

**Figure 7:**
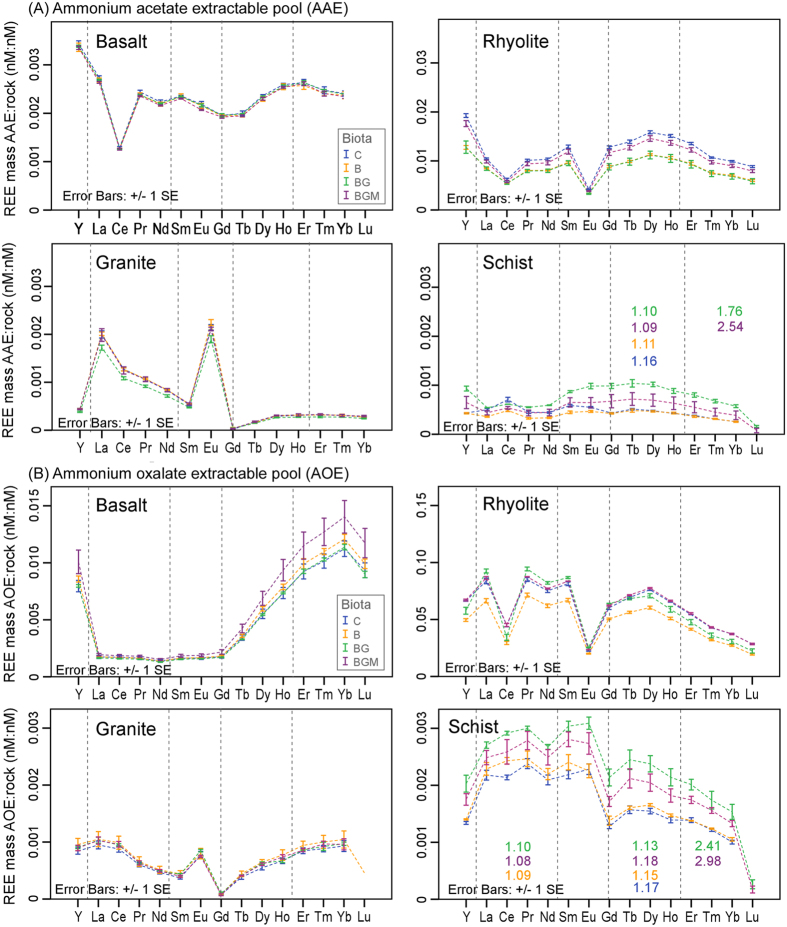
REE variability in easily extractable solid pools. Rock-normalized REE abundances in (**A**) ammonium acetate (AAE) and (**B**) ammonium oxalate (AOE) rock extracts at the end of the 20-month experiment as affected by the different treatments. Values are means of total REE mass (nmol, nM) extractable pool: initial rock in each column (n = 3). Tetrads are grouped by vertical lines with tetrad value colors representing treatment color.

Sequential chemical extraction of water soluble, carbonate and exchangeable (ammonium acetate extractable, AAE) pool (Fig. 7A) showed a biota effect that was opposite to changes in the solution phase (Fig. 3). Since this fraction can be considered bioavailable, the observed effect can be partially attributed to plant uptake.

In rhyolite and granite (both felsic), biota caused a decrease in the exchangeable (AAE) pool, particularly in the BG treatment, coinciding with a comparatively well-developed plant biomass (Figure s4). These differences were significant at 95% for most elements in rhyolite. In schist, an increase in the AAE pool under vascular plant relative to microbial and control treatments (significant at 95% for most REE in BG treatment) coincided with the smallest plant biomass (Figure s4).

Mycorrhiza addition increased this pool compared to grass-only in rhyolite, and decreased it in schist, mirroring the aquatic effect (Fig. 3). Results from the first four months of the experiment showed that major nutrient mobilization (denudation and uptake in the vascular plant) is governed by element supply from parent mineral and plant physiological requirements^53^. Therefore, given similar biotic consortia in all rocks, the observed biotic differences in exchangeable pools among substrates must be directly connected to biological response to major nutrient balances of each rock. Except for basalt and rhyolite, which developed Ce anomalies, probably due to conditions unfavorable for its redox transformation, the general patterns across REE series mimicked their water counterparts (Fig. 3).

Analysis of the more stable poorly-crystalline (ammonium oxalate extractable, AOE) pool (including precursors of secondary minerals, such as hydrous oxides) revealed that generally, Y and heavier REE preferentially precipitated in basalt, whereas lighter REE precipitated in rhyolite and schist (Fig. 7B). A rhyolite Ce anomaly resembling one observed in AAE, as well as Eu anomalies in granite and rhyolite found in water and AAE were also translated to this pool. With few exceptions, biotic treatment effects were complex (rock specific), and broadly resembled those observed in AAE. Specifically, microbes significantly (*p* < 0.05) decreased REE retention in the AOE pool (as compared to control) in rhyolite (Fig. 7B). Vascular plant treatments increased the retention over microbe treatments in schist and rhyolite; and mycorrhiza increased REE retention in the AOE pool in basalt (*p* < 0.05 for most REE) and decreased it in schist (*p* < 0.32). In rhyolite, mycorrhiza generally decreased L-REE and increased H-REE retention (*p* < 0.05 for most REE).

An M-type tetrad effect (expected for sediment/soil)^54^ developed in schist in both AAE and AOE pools (Fig. 7). The first tetrad in AOE appeared only in biota systems (Fig. 7B). Since our study only covers a short period, it is expected that over longer weathering, or in natural soil genesis setting the amplitude of the effect will be clearer (larger), and it would develop on a multitude of substrates. Results of REE measured in pore water, exchangeable and poorly crystalline pools reveal that in newly established ecosystems or during incipient soil formation from rock, there is a significant biotic regulation of REE distribution among different biogeochemical pools, and this is particularly evident in the vegetated treatments.

### Coupled denudation, uptake, and stabilization in secondary solid phases

**Figure 8:**
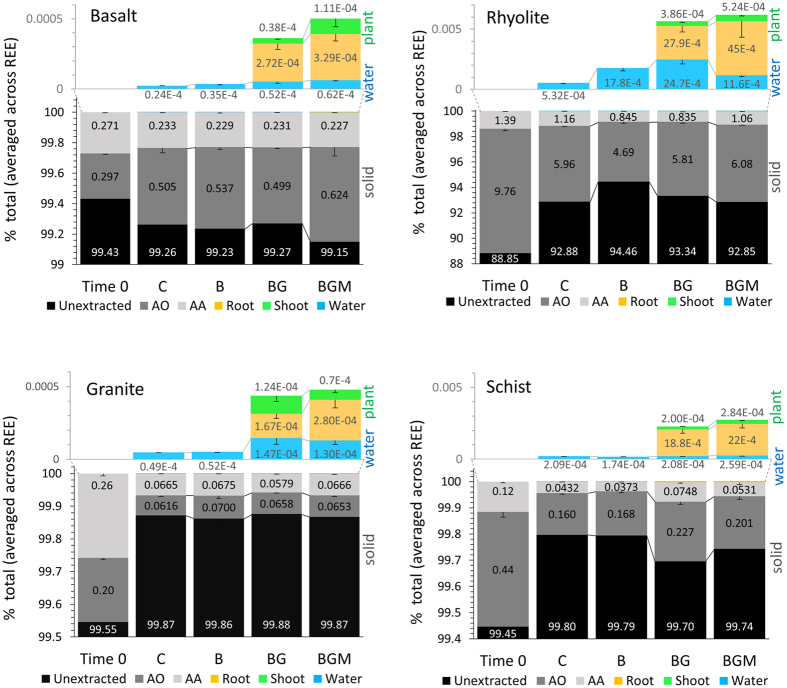
Mass balance of abiotic and biotic REE weathering. Distribution of sum of REE at time = 0 and the end of the 20-month experiment as a function of rock type, abiotic and biotic treatments (C, control; B, bacteria; BG, grass-bacteria; BGM, grass-bacteria-mycorrhiza). Shown REE pools include unextracted (black), ammonium oxalate extractable (dark gray), ammonium acetate extractable (light grey), water denudation (blue), root biomass (ochre) and shoots biomass (green). Values are means of column triplicates. For each column values were summed across REE series, and for water also across sampling events. Error bars represent +/−1SE.

Microbial and plant communities are critical players in elemental balance in the environment by mobilizing, accumulating and redistributing elements originating from weathering rocks. Quantitative mass balance analysis showed the largest total REE mobilization (solution and plant) in rhyolite, followed by schist, granite, and basalt (Fig. 8). The extractable (more bioavailable) solid pools (AAE and AOE) ranged from almost 10% of rock total in rhyolite to <1% in basalt and <0.1% in granite and schist. They were two to three orders of magnitude larger than the mobilized (water + plant) pool. However, the mass ratio of extractable to mobilized REE decreased from C to BGM (Table s5), which implies an incremental effect of ecosystem complexity on REE mobilization-sequestration balance in all rocks.

The biological effect on mobilization from rock was significant (*p* < 0.05), the difference being caused by both changes in denudation (dissolved forms) and the contribution of bio-uptake. Un inoculated controls, representing baseline abiotic weathering generally showed the lowest REE denudation, while the microbial community enhanced the denudation, with significant effects in rhyolite. This coincided with decreased exchangeable (AAE) and poorly crystalline (AOE) pools in this rock.

Plant and microbial presence significantly increased denudation over control, from 2.2 times in basalt to 3.0 in granite and 4.6 in rhyolite. As plant biomass accumulated a greater amount of REE than denudation, this result indicates that the development of vascular plants significantly increased total REE mobilization (in solution and plant) in all rocks by about an order of magnitude relative to the abiotic control (Fig. 8). Most of the plant REE were stored in the root, reflecting a high REE weathering efficiency of the root-microbe consortium. In rhyolite and schist, where plant biomass was higher as compared to the other rocks (significant for rhyolite at 67% level), plants induced an increase in poorly crystalline + exchangeable pools. The activity of arbuscular mycorrhiza generally increased REE mass in plant tissue by 1.2 to 1.6 times (*p* < 0.05), but in rhyolite an increased plant uptake was balanced by decreases in dissolved forms (*p* < 0.05). As a result, total mobilization was not significantly affected by mycorrhiza in rhyolite, whereas exchangeable and amorphous pools were shown to increase.

It is also noteworthy that both, exchangeable and amorphous pools were relatively large in the initial rock (Fig. 8). We attribute this to the initial reactivity of fresh mineral surfaces wherein crystal structures are exposed by rock grinding and microfractures readily release elements to chelating agents. The large extractable pool in the unreacted rock and the initial high spike in denudation (Fig. 2) suggests that the weathering rate is limited by the prevalence of reactive surfaces. Microfragments from abiotic fracturing, together with microbes, lysed border cells released from growing root tips^55^ and complexing agents secreted by roots and microbes, together may form incipient soil. Given that a significant biotic effect was observed in AAE and AOE pools (Fig. 6), it is safe to assume that a portion of them represents novel exchangeable and amorphous phases resulted from weathering, which contribute to soil formation. Further instrumental analysis of the solid products of weathering, the focus of future research, will shed more light on the composition of incipient soil.

The results suggest that in the transition from simple to complex, e.g. in ecosystem colonization of freshly exposed bedrock including fresh volcanic fields, mountain tops, fresh surfaces exposed by glacial retreat, and fresh bedrock introduction into the bottom of the critical zone weathering profile, biota play a major role in initiating REE cycles. Secondly, since roots of vascular plants have taken up a significant mass of weathered REE, they are good sensors, integrators and regulators of REE cycle in the biosphere.

### Global REE weathering estimates

Using current data on global weathering estimates for major elements derived from river fluxes (lithology-specific) and water elemental stoichiometry in the experiment (SI 1.3), we estimated a total annual REE denudation rate of about 3859*10^4^ moles from the four exposed lithologies. Of this, 60*10^4^ moles are contributed by basalt, 924*10^4^ moles by rhyolite, 933*10^4^ moles by granite and 1942*10^4^ moles by schist. Abiotic weathering alone contributes about 39 ± 19% of these values (12.4 basalt, 48.5 rhyolite, 30.4 granite, and 63.1 schist, % of GBM), with the remaining added by the biological component of the ecosystem. Given that solute denudation to oceans is dominated (>50%) by the 10% of Earth’s surface exhibiting high relief, i.e. mountains^56^, we assume that most REE weathering is likewise restricted to such regions, particularly those where rock is being actively transformed to soil, comparable to our experiment. However, these estimates are clearly a first-order approach to understanding global REE fluxes.

## Conclusions

A bio-weathering experiment conducted for two years with four rock types using REE as tracers, and wherein nutrients were derived solely from rock resulted in sustained biotic development. The experiment indicated that earliest weathering progressed rapidly, with little difference between abiotic and biological treatments—mineral dissolution being dominated by abiotic processes and enhanced by physical microfracturing. However, in approximately two months leaching rates declined while rock-colonizing microorganisms and plants began to exert greater influence of leaching and uptake, and this ultimately dictated REE distribution and fate. Results further indicated that inorganic dissolution enhanced by biotic respiration was predominant throughout the experiment, consistent with low organic matter presence.

The REE partitioning between water, plant and secondary solid-phase pools (exchangeable and poorly crystalline) varied slightly with rock type, but several consistent trends were observed. At the end of the experiment, ten times more REE had been mobilized in planted compared to abiotic treatments, with plants hosting a significantly larger REE pool than that released to water alone. Roots stored greater REE mass compared to shoots due to both greater biomass and higher REE concentration, meaning that below ground biomass is the dominant biological store of REE in the ecosystem. Comparatively lower shoot concentrations can be ascribed to REE retention with P and other major ions in root endoderm’s cell walls.

Total REE mobilization (leaching plus plant uptake) was generally increased by arbuscular mycorrhiza, but in rhyolite decreased leaching losses were counteracted by greater retention in the exchangeable pool. This type of symbiosis, therefore, significantly contributes to REE cycles by controlling REE export and retention balances. Microbes, while having a smaller effect on REE denudation and total mobilization than plants, resulted in significant increases in both parameters in rhyolite, in association to lower masses of bioavailable solid pools. The results also imply that more complex ecosystems are generally capable of preferentially mobilizing, accumulating and redistributing larger masses of REE in the environment than simpler ones, even early in the biological colonization. This was also supported by extractable solid-phase pools, which decreased relative to the dissolved and plant pools as treatment complexity increased.

A synergetic interplay between rock, water, and biota determined variability patterns among REE, with both rock mineralogy and biotic system imprinting differences among these patterns. REE in biota can therefore directly reflect soil genesis, including rock weathering/denudation capacity. The similarity in REE patterns detected between root and shoot makes the above-ground biomass a reliable integrator and sensor of REE in the belowground ecosystem. Most importantly, there was a strong REE signature of bio-weathering, reflected by the weathering magnitude, the general REE pattern (particularly L-REE), the development of a tetrad effect, a consistently increased REE mobilization-sequestration ratio with ecosystem complexity, and a consistent REE:phosphate BSI index in water for all rock types. This shows that selective REE interaction with the ecosystem exists, and this can drive element partitioning during bio-weathering, leaving detectable and persistent signals of life in both effluent waters and weathered media.

Our results provide a quantitative understanding of biological contribution to REE weathering in common upper crust rocks. This can have broad implications in modeling their global cycles and budgets in relationship with overlaying ecosystems, the future environmental changes, as well as for understanding the potential biological signatures accumulated in Earth’s historical record, and on other planetary bodies.

## Methodology

### Experimental design and solution analysis

A model ecosystem experiment was setup in the Desert Biome at the University of Arizona’s Biosphere 2 (Figure s1). Four granular substrates (basalt, rhyolite, granite and schist) and six ecological treatments were placed in 288 experimental columns (30 cm long × 5 cm internal diameter) in six controlled-environment chambers (170 cm length × 0.77 cm width × 0.98 cm height) receiving filtered and UV light-sterilized air and purified water. For this study, we report the effect of four biological treatments on the weathering of the four bedrock types during the first 20 months of colonization. In increasing order of complexity, treatments were: abiotic control (with autoclaved microbial consortium) (labeled as C); microbes (a natural consortium extracted from basalt collected in Merriam crater, Flagstaff, AZ) (labeled as bacteria, B); microbes and grass (Buffalo grass, *Bouteloua dactyloides*) (BG); and microbes, grass and mycorrhiza (grass infecting *Rhizophagus irregularis*) (BGM). Treatments were run in triplicate with water samples collected over 21 time points (December 2012–July 2014). To allow solid and biotic phases analysis over four time points (at 132, 252, 465 and 584 days), treatments were repeated four times.

The substrate materials were collected from Santa Catalina Mountains (medium-grain Oracle granite and micaceous Pinal schist); Meriam crater, Flagstaff, Arizona (cinder basalt); and Valles Caldera National Preserve, New Mexico (rhyolite), U.S.A. These rocks were derived from the Catalina-Jemez Critical Zone Observatory and the Biosphere 2 Landscape Evolution Observatory, two related large-scale experiments that study the coupled chemical, hydrological, and ecological processes that shape the Earth’s surface and support most terrestrial life^2^,^57^. Except for basalt (fresh tephritic material of very limited weathering, which was ground at the mining site), the rocks were subjected to mechanical removal of all weathered crust, before being crushed and ground in jaw and roll crushers. All substrates were then dry and wet sieved to 250–500 μm particle size, passed on a Wilfley water table to remove impurities, and rinsed several times with nanopure-grade water. The clean material was then dried at 85 °C. Substrate characteristics are in Table s1. The rock was then sterilized by autoclaving for one hour over 3 consecutive days. Sterilization was confirmed by a lack of colony-forming bacterial growth on R2A agar plates.

Each column received 90 mL of inoculum containing 1.43 × 10^5^ colony forming bacteria per mL under sterile hood, incubated at room temperature for one week in sterilized jars, packed to top of the column in UV-light sterilized acrylic columns (Table s1 for rock weight) and planted with dehusked and pre-sterilized germinated grass seeds (20 per column, 2 cm depth; purchased from Western Native Seed, Colorado, USA)^53^. Control samples were inoculated with a sterilized inoculum to retain a consistent chemical elemental composition. A 1-mL water suspension of pure *R. irregularis* spores and mycelium (about 400 spores; Premier Tech Biotechnologies, Canada) were added to each mycorrhizal treatment column next to the seedling.

A sterilized 140 mL polypropylene syringe (Nasco, Modesto, CA) was used to add 100–120 mL of sterile nano-pure water (18 MΩ) per column at room temperature every two weeks to support the bioreactor function. About 35–50 mL sample was collected gravimetrically biweekly for the first two months and monthly thereafter. This allowed for complete flushing of substrate pore space at sampling, while unflushed columns achieved about 50% column saturation depth, dropping to 0% by the next watering/sampling event. The irrigation system was designed to prevent preferential water flow in the substrate (Figure s1). Water samples were analyzed for pH, electrical conductivity (by electrode), total organic carbon (680 °C combustion catalytic oxidation, Shimadzu TOC-L system) and major, trace and REE by inductively coupled plasma mass spectrometry (ICP-MS, Perkin Elmer, Elan DRC-II). Major anions in solution, i.e. fluoride, chloride, nitrite, bromide, nitrate, sulfate and phosphate were analyzed by ion chromatography (Dionex). The water balance was determined for each column by subtracting water output volume from water input at the time of sampling, and it is assumed to indicate substrate water use capacity, i.e. retained and transpired by biota, and evaporated by column surface.

### Initial rock characterization

Mineralogical characterization was performed on all rock samples (250–500 μm size fraction) by electron microprobe and X-ray diffraction. Microprobe data were collected using CAMECA SX100 Ultra and CAMECA SX50 electron probe microanalyzers (Lunar and Planetary Sciences Laboratory, University of Arizona, USA). A beam size of 5 μm (15KeV, 20 nA) was chosen to probe chemical composition in feldspars and glass and 1μm for other minerals. For the calculation of chemical formulae, the elemental composition was normalized to oxygen. In addition, 5 × 5 mm elemental maps were collected to characterize element heterogeneity of the sample, rock microstructure, and to provide additional means of determining mineral composition. High current mode (25 KeV and 20 nA) was used for identification and mapping of selected REE and their mineral hosts.

To quantify mineral abundance, samples were micronized to <2 μm and subjected to high energy X-ray diffraction (XRD) on beamline 11.3 at the Stanford Synchrotron Radiation Lightsource (SSRL), USA. The line was operated in transmission mode at ca. 12735 eV, using a 34.5 cm radius Mar detector with 100 μm pixels. Three scans were collected for each 0.05 g sample and combined. Quantitative analysis of minerals was performed using the Rietveld module included in the X’Pert HighScore Plus software. Total elemental composition of rock samples (same element set as in water) was measured following lithium/tetraborate fusion followed by ICP-MS analysis (Activation Laboratories Inc., Ancaster, Ontario, Canada).

### Plant material analysis

After 20 months, columns were sealed under sterile conditions, extracted from growth chambers, and stored at 4 °C until lab processing. Under a laminar-flow hood, plants were separated from loosely attached rock, washed with nanopure-grade water to remove remaining particles, dried at 70 °C for 3 days, separated into above and below ground biomass, and weighed. Subsamples of above and below ground biomass were microwave digested in 1:1 70% HNO_3_: 30% H_2_O_2_ mixture at 200 °C. REE concentrations were measured by ICP-MS together with the same suite of major and trace elements. Standard quality control measures including sample blanks, certified reference material (apple leaves CRM 1515) and triplicate sample analysis were implemented for each digestion batch.

### Sequential extraction

Following removal from the column, bulk geomedia was air dried, homogenized, subsampled, and subjected to a two-step sequential chemical extraction following a modified protocol from ref. 58. The procedure extracted (a) soluble, exchangeable ions and carbonates (using 0.2 M ammonium acetate adjusted to pH 4.5) and (b) amorphous-to-poorly crystalline pool (using 0.2 M ammonium oxalate adjusted to pH 3.0 with 0.2 M oxalic acid).

### Data analysis

To understand the broader context of substrates used, REE abundances in the four rocks were analyzed together with their terrestrial upper continental crust values^33^, mantle and carbonaceous chondrite (protoplanetary material)^59^ by Principal Component Analysis (PCA). PCA is a statistical method, which can reduce a great number of variables to few composite variables (principal components) representing major trends associated with the dataset. Rock concentrations were then normalized by upper continental crust average values to identify potential REE enrichment/depletion. PCA was further used to identify associations between rock-normalized REE, major and trace element abundances in plants, and between REE and major elements in sequentially extracted phases.

ANOVA with Fisher’s least significant difference (LSD) posthoc test of inter-treatment comparisons was used to test the effect of mycorrhiza on plant REE uptake, and the effect of various biotic components of ecosystem on total REE mass distribution among weathering pools in a mass balance analysis. Preferential uptake was assessed by dividing total element in the plant (mass) to total removal/leaching in water (mass).

The tetrad effect was identified by visual inspection of tetrad shapes in bulk rock-normalized concentrations in water and sequential extraction materials, and water-normalized plant tissues. The effect size (t) was determined for each of the four tetrads (i) using the Eq. (1) 40, which is the equivalent of the method proposed by ref. 60.


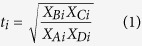


where *X*_*Ai*_ and *X*_*Di*_ are the normalized concentrations of first and last tetrad members, and *X*_*Bi*_ − *X*_*Ci*_ is the normalized concentrations of the central elements. The formula compares the center members with an imaginary line that connects the first and last members in a logarithmic plot. The values of t_i_ are larger than one for convex (M-class) curves, equal to one for a straight line (no tetrad), and <1 for concave (W-class) curves. Tetrads displaying Ce and Eu anomalies were not quantified. Due to a typically high intrinsic (measurement) error in REE quantification, inter-treatment comparisons were on ±1 standard error (SE). Statistical analyses were computed in IBM-SPSS statistics v.21.

## Additional Information

**How to cite this article:** Zaharescu, D. G. *et al*. Ecosystem Composition Controls the Fate of Rare Earth Elements during Incipient Soil Genesis. *Sci. Rep.* **7**, 43208; doi: 10.1038/srep43208 (2017).

**Publisher's note:** Springer Nature remains neutral with regard to jurisdictional claims in published maps and institutional affiliations.

## Supplementary Material

Supplementary Information
