# Supplementary material for: Ecosystem Composition Controls the Fate of Rare Earth Elements during Incipient Soil Genesis
Source: Sci Rep. 2017 Feb 23;7:43208. doi: 10.1038/srep43208 (PMC5322375; doi:10.1038/srep43208)
Supplement: Supplementary Information [file srep43208-s1.pdf]

Ecosystem Composition Controls the Fate of Rare Earth Elements during Incipient Soil Genesis

Dragos G. Zaharescu[a,b(*)], Carmen I. Burghelea[a], Katerina Dontsova[a,b], Jennifer K. Presler[a], Raina Maier[b], Travis Huxman[c], Kenneth J. Domanik[d], Edward A. Hunt[a], Mary K. Amistadi[b,e], Emily E. Gaddis[a,f], Maria A. Palacios-Menendez[a,g], Maria O. Vaquera-Ibarra[a,h], Jon Chorover[a,b]

[a] Biosphere 2, The University of Arizona, Tucson, AZ, USA
[b] Department of Soil, Water & Environmental Science, The University of Arizona, Tucson, AZ, USA
[c] School of Biological Sciences, University of California, Irvine, CA, USA
[d] Lunar and Planetary Laboratory, The University of Arizona, Tucson, AZ, USA
[e] Arizona Laboratory for Emerging Contaminants, The University of Arizona, Tucson, AZ, USA
[f] Williams College, Williamstown, MA, USA
[g] The University of Caribe, Cancún, México
[h] University of the Americas Puebla, Puebla, México

SI 1 Supplementary Methodology

SI 1.1 The experiment

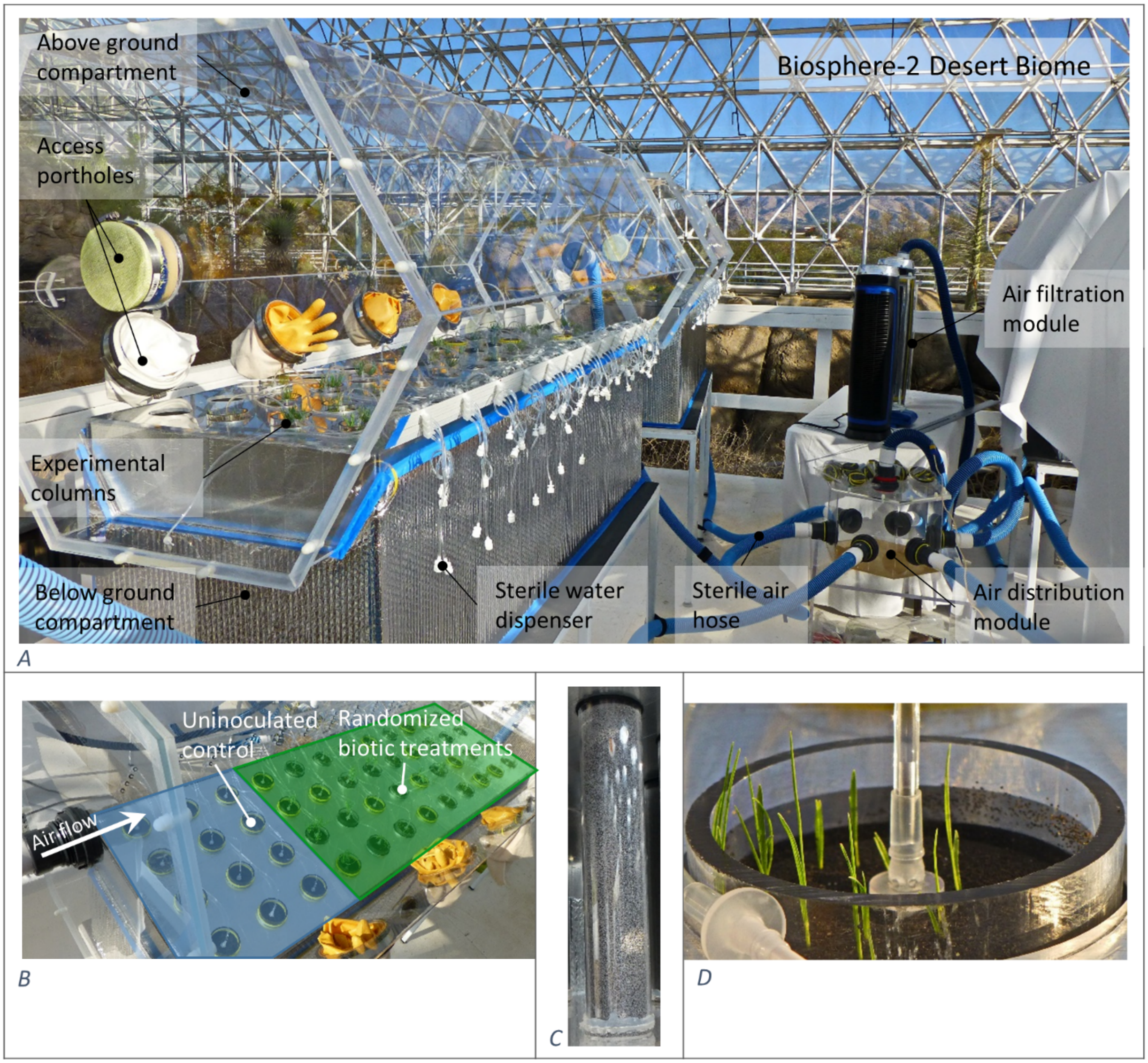

Figure s1. **Experimental mesocosms in the Desert Biome of Biosphere-2** (A) containing granular rock-filled columns and purified air and water delivery systems. (B) Treatment distribution in the modules with un-inoculated control first in the direction of air flow. (C) Example of experimental column filled with granular granite. (D) Two weeks old Buffalo grass growing in granular basalt, with water dispenser designed to avoid preferential water percolation in the substrate.

A model ecosystem experiment was setup in the Desert Biome at University of Arizona's Biosphere 2 based on a design detailed in ([1,2]). Briefly, 6 enclosed chambers connected in parallel to a double air purification system (using 2 high-efficiency particulate absorption HEPA filters and 2 UV-B air sterilization light sources, capable of delivering about 1L air sec^{-1} per module; Germguardian, AC4850CAPT Digital 3-in-1 Hepa Air Purifier System) hold 288 experimental columns (30 x 5 cm internal

diameter; Figure s1). Except for control columns, which were placed first in the direction of air flow, the columns were grouped by rock type and randomly distributed in each module. The temperature in the modules/experiment followed the one in the Desert Biome, which was kept at a mean temperature of 19±4°C, relative humidity of 48±19%, and natural O_2/CO_2 saturation conditions. Modules' aerial chambers experienced additional solar radiative heating of about 5°C above the Biome average during the day. Belowground (soil) compartments were light and thermally shielded, which prevented overheating.

SI **1.2 Biological signature index**

To infer a biological signature index, the following lines of evidence were considered: (a) REE sources in phosphate minerals, REE-oxides, and minor minerals (ilmenite, titanite, zircon, allanite) in the used rocks; (b) REE mobilization under biotic treatment was radius dependent, L-REE exhibiting increased mobilization under biota; and, (c) P is the mineral constituent of principal biotic relevance. Based on these premises we propose using an abiotic control-normalized ratio of La : phosphate water concentrations as biotic signature index, following the equation *s1*.

$$BSI = 100\frac{x - min(x)}{\max(x) - \min(x) - Z} + 1 \qquad \text{(E. } s1\text{)}$$

$$\text{Where, } x = Log\frac{1}{100 - \frac{La}{Phosphate}}$$

Where, the term 100(*x*-min(*x*))/(max(*x)*-min(*x)*) represents a 0-100 scale normalization. *X* is a Log-normalization ratio of La:Phosphate concentrations, and Z, a rock-specific constant, representing the abiotic fraction of *x*. Based on water concentration data of our control treatment Z takes four values (mean±SE): Z_{Basalt}=0.543±0.124; $Z_{Rhyolite}$=1.53±0.339; $Z_{Granite}$=0.626±0.384; and, Z_{Schist}=1.23±0.315.

SI **1.3 Global denudation estimates**

Estimated values for global (G) REE denudation rates (moles * year^{-1}) were inferred by stoichiometric adjustment of Na- normalized total REE in our experiment (i) to global Na values from river data[3], according to equation *s2*.

$$\sum\nolimits_{REE_G} = \frac{\sum_{REE_i}}{Na_i} Na_G \qquad \text{(E. } s2\text{)}$$

To infer REE contribution by different rocks, the global Na value from rivers was adjusted (multiplied) to the relative contribution (%) of the rock to the global exposed lithology described in ([4]). Abiotic and biotic contribution to the global cycle was estimated from their ratio in the experiment.

SI 2 Supplementary Results

SI **2.1 Substrate characterization**

SI 2.1.1 Rocks chemistry in the global context

Multivariate analysis of rock REE abundances showed greater similarity of the used substrates with the upper (basalt, rhyolite and granite) and lower (schist) terrestrial crust (> 80%variability in dataset), than with the upper mantle and protoplanetary material (carbonaceous chondrite) (Figure s2). Therefore, REE abundances in the substrates were not exceptions to average values of terrestrial crust.

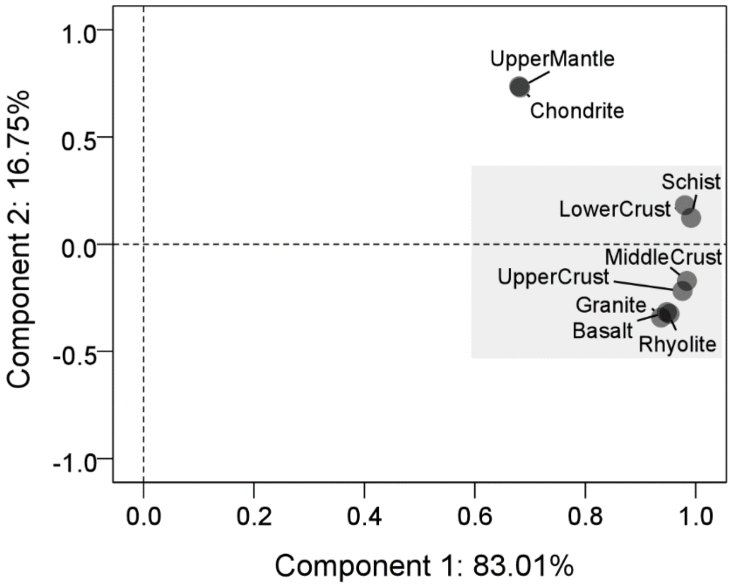

Figure s2. **Studied rocks in the context of terrestrial and protoplanetary material.** Samples were associated based on their similarity in REE content by Principal Component Analysis. Shaded in grey are data strongly associated to component 1 that explains most of the variability in the dataset.

SI 2.1.2 REE mineral source

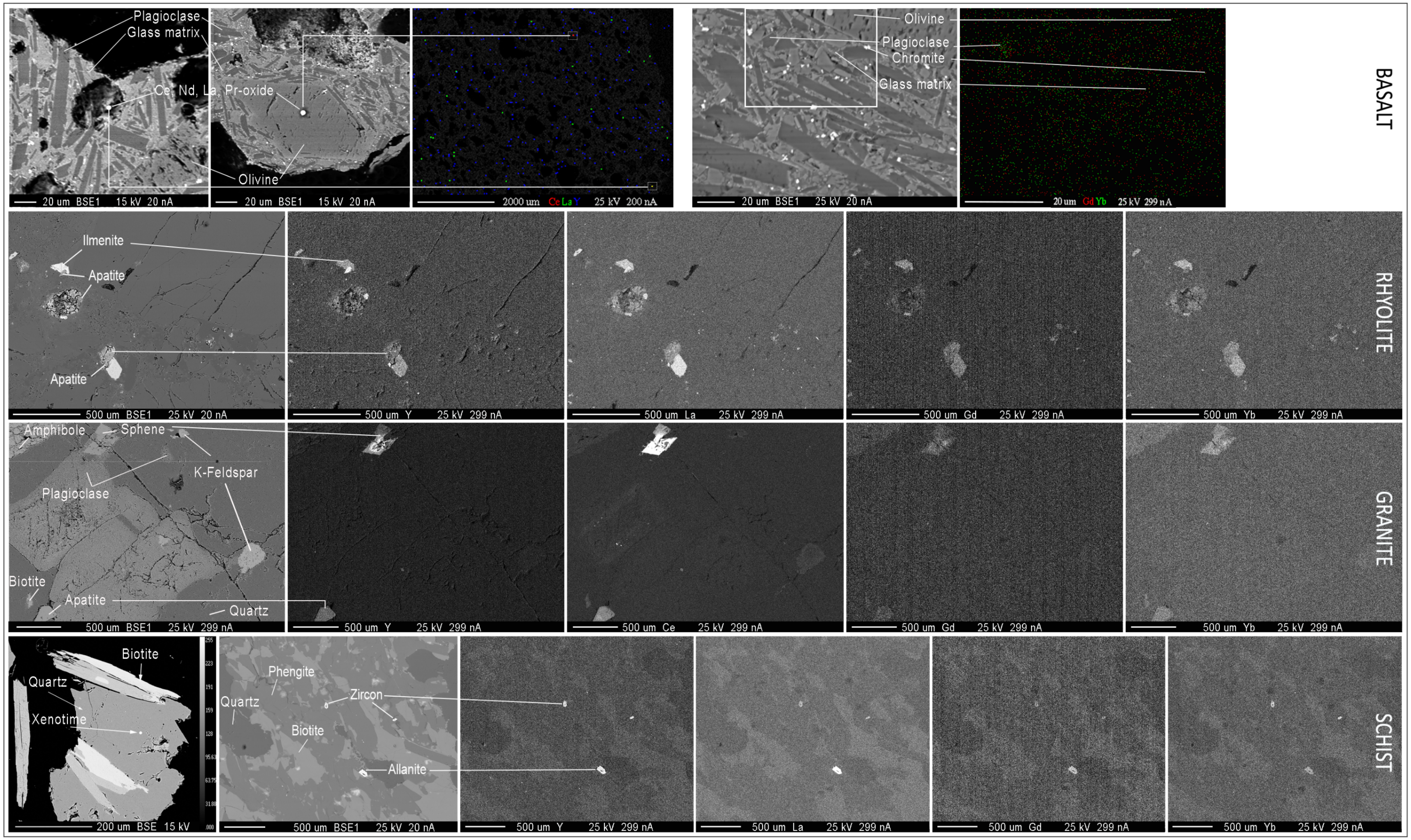

Figure s3. **REE mineral source.** Back scatter images of studied rocks and high current electron microprobe maps of representative L-, M- and H-REE in the studied materials, and the limited number of minerals that host them. Scattered X-rays appear brighter relative to background (increased contrast) with increasing REE density. Ce-Nd-La-Pr oxide in basalt, and allanite and xenotime in schist have been identified as main REE mineral hosts. Pixel size in multi-element maps has been exaggerated 4X to improve perception of otherwise very low levels.

SI 2.1.3 Substrate physical and mineral characteristics

Table s1. **Substrate characteristics** (expanded from [2]). Mineral formulas were calculated from electron microprobe elemental analyses (a mean of several point analyses), and mineral abundances were estimated quantitatively by Rietveld analysis of X-ray diffraction data. Table headers contain additional information on physical properties of substrates used in the experiment.

**Basalt**	690 ± 6 g column^{-1} Density: 1.32± 0.03g cm^{-3} Pore volume: 33± 0.7%	**Average %**
Volcanic glass	$Ca_{0.36}Mg_{0.22}Na_{0.16}K_{0.09}Mn_{0.01}Fe_{0.49}Al_{0.58}Ti_{0.11}(HPO_4)_{0.05}Si_{1.87}O_{5.89}$	NA
Andesine		50.5
Augite	$Ca_{0.86}Na_{0.03}Mg_{0.78}Fe^{2+}_{0.27}Al_{0.22}Ti_{0.05}Si_{1.81}O_6$	21.8
Forsterite	$Mg_{1.55}Fe_{0.35}(Si_{1.04}O_4)$	21.2
Labradorite	$Na_{0.32}Ca_{0.68}Al_{1.56}Si_{2.35}O_8$	3
Titanomagnetite	$Fe_{2.19}Ti_{0.55}Mg_{0.21}Al_{0.15}Si_{0.11}O_4$	2
Minor minerals:	Chromite (*$(Fe^{2+}_{0.66}Al_{1.24}Mg_{0.64})Cr_{0.51}O_4$), Apatite	< 1 each
**Rhyolite**	690 ± 10 g column^{-1} Density: 1.24± 0.02g cm^{-3} Pore volume: 35± 1%	
Oligoclase	$Na_{0.69}Ca_{0.24}Al_{1.23}Si_{2.76}O_8$	36.7
Sanidine	$K_{0.36}Na_{0.54}Al_{1.02}Si_{2.99}O_8$	22.8
Quartz	SiO_2	17.2
Anorthoclase	$Na_{0.70}K_{0.23}Al_{1.05}Si_{2.94}O_8$	16.7
Ilmenite	$Fe^{2+}_{0.82}Ti_{1.02}O_3$	5.0
Titanite		1.5
Minor minerals:	Titanohematite ($Fe^{3+}_{2.49-2.52}Ti_{0.1-0.15}O_3$), Faujasite, Phengite, Apatite, Zircon	< 1 each
**Granite**	640 ± 8 g column^{-1} Density: 1.34±0.003g cm^{-3} Pore volume: 32± 0.8%	
Albite	$Na_{0.95}Ca_{0.003}Al_{1.02}Si_3O_8$	32.3
Sanidine	$K_{0.86}Na_{0.15}Al_{0.98}Si_3O_8$	18.5
Oligoclase	$Na_{0.73}Ca_{0.24}Al_{1.18}Si_{2.78}O_8$	18.4
Quartz	SiO_2	18.0
Biotite	$K_{0.91}Mg_{1.56}Fe^{2+}_{1.06}Al_{1.15}Si_{2.83}O_{10}(OH)_{1.75}F_{0.25}$	6.6
Titanite	$Ca_{1.04}Ti_{0.77}Al_{0.25}Fe^{3+}_{0.04}SiO_5$	3.7
Apatite	$Ca_{5.22}(PO_4)_3F_{0.23}$	2.3
Minor minerals:	Muscovite ($K_{1.24}Al_{1.34}Si_{4.14}O_{10}(OH)_2$), Chlorite ($Mg_{2.73}Fe^{2+}_{1.84}Si_{2.96}Al_{2.25}Mn_{0.12}O_{10}(OH)_8$), Ilmenite ($Fe^{2+}_{0.74}Ti_{0.96}Mn_{0.32}O_3$), Garnet ($Ca_{2.38}Fe^{3+}_{0.83}Ti_{1.59}Al_{0.63}Si_{2.33}O_{12}$), Magnetite	< 1 each
**Schist**	480 ± 5g column^{-1} Density: 1.04± 0.02g cm^{-3} Pore volume: 43± 1%	
Quartz	SiO_2	47.9
Muscovite		21.8
Phengite	$K_{0.87}Al_{2.47}Fe_{0.21}Mg_{0.18}(OH)_2Si_{3.20}O_{10}$	19.7
Biotite	$K_{0.94}Mg_{1.66}Fe^{2+}_{0.88}Al_{1.40}Si_{2.87}O_{10}(OH)_{1.57}F_{0.43}$	10.1
Minor minerals:	Xenotime, Zircon, Allanite	< 1 each

Basalt consisted of amorphous glass matrix incorporating andesine, olivine and pyroxene, as dominant phases, with traces of other minerals (Table s1). It is higher in Ca, Mg and Fe, and lower in Si than other studied rocks. The vesicular structure of basalt suggested comparatively faster weathering potential. Rhyolite was rich in feldspars and quartz (Table s1). The geochemistry of rhyolite was similar to granite, but it was richer in Si and Na and had less Ca ([2]). Schist contained a high proportion of Mg-rich phengite, a transitional phase between muscovite and caledonite.

SI 2.2 Major pore-water descriptors

*Table s2***.** Total REE, Sr (major weathering indicator, for comparison), total organic carbon (TOC), water balance - representing the water used by biota (transpired and tissue-stored) + column evaporation (expressed as difference between input and output volumes) and mean measured pH and electrical conductivity (EC) over the 20-months experiment. Mycorrhiza infection rates are also presented for each substrate.

Rock<br>(% infection±SD)	Treatment	N	REEsum (ng)<br>Mean ± SD	Sr (ug)<br>Mean ± SD	pH<br>Mean ± SD	EC (µS/cm)<br>Mean ± SD	TOC (mg)<br>Mean ± SD	Water balance (ml)<br>Mean ± SD
**Basalt**	C	3	13.27 ± 1.25	73.78 ± 4.26	8.03 ± 0.02	167.1 ± 5.47	39.69 ± 3.21	1039 ± 61.63
(67±18)	B	3	47.29 ± 18.88	88.61 ± 11.00	8.01 ± 0.01	190.5 ± 22.32	23.65 ± 7.68	939.0 ± 73.04
	BG	3	62.92 ± 31.78	113.7 ± 20.25	8.01 ± 0.05	228.3 ± 19.29	17.27 ± 0.97	1053 ± 41.99
	BGM	3	88.11 ± 12.33	124.4 ± 24.97	7.99 ± 0.03	249.7 ± 1.83	12.72 ± 1.66	954.0 ± 33.45
**Rhyolite**	C	3	516.3 ± 268.5	61.77 ± 12.21	7.80 ± 0.05	134.9 ± 12.32	12.27 ± 2.80	990.7 ± 34.35
(52±30)	B	3	1748 ± 321.7	71.19 ± 11.56	7.61 ± 0.05	123.9 ± 17.97	34.75 ± 4.65	856.0 ± 112.6
	BG	3	2569 ± 654.2	71.52 ± 14.47	7.64 ± 0.07	127.3 ± 14.43	17.96 ± 0.74	844.3 ± 52.14
	BGM	3	1141 ± 176.0	71.13 ± 1.51	7.77 ± 0.06	158.0 ± 21.21	13.96 ± 4.18	813.6 ± 76.34
**Granite**	C	3	69.26 ± 4.84	152.9 ± 5.77	8.04 ± 0.03	211.6 ± 8.41	28.60 ± 8.12	944.8 ± 34.69
(0±0)	B	3	75.85 ± 11.76	134.2 ± 8.57	8.07 ± 0.02	198.9 ± 5.54	22.02 ± 3.35	1058 ± 15.10
	BG	3	262.5 ± 144.2	173.7 ± 18.07	8.06 ± 0.04	265.9 ± 1.74	44.37 ± 23.86	1006 ± 198.6
	BGM	3	224.5 ± 102.1	181.5 ± 28.21	7.99 ± 0.06	267.7 ± 35.38	40.62 ± 21.89	972.6 ± 125.8
**Schist**	C	3	144.3 ± 32.74	35.72 ± 4.70	6.27 ± 0.40	59.23 ± 9.96	34.69 ± 9.04	2195 ± 99.52
(18±20)	B	3	128.6 ± 23.96	18.41 ± 1.70	7.06 ± 0.09	41.92 ± 3.71	28.84 ± 6.56	2270 ± 81.37
	BG	3	135.6 ± 32.40	33.70 ± 6.89	7.24 ± 0.08	56.48 ± 3.12	20.60 ± 2.33	1980 ± 25.38
	BGM	3	179.3 ± 102.4	21.68 ± 3.66	7.06 ± 0.14	39.75 ± 8.33	26.19 ± 7.42	1817 ± 22.60

SI **2.3 Co-dissolution and uptake mechanisms**

SI 2.3.1. Water

There was a strong correlation among concentrations of different REE in pore water (Table s3) consistent to their group behavior.

Table s3. Principal Component Analysis heat map showing correlations between water concentrations of REE, major elements in their mineral sources, total organic carbon (TOC) and other major constituents (pooled across all samples and columns), that can explain their dissolution mechanisms. Values are arranged in decreasing order of correlation with each component. For easy identification REE are in bold.

Water												Color code	-1	0	1
**Basalt**	PC 1	PC 2	PC 3	**Rhyolite**	PC 1	PC 2	PC 3	**Granite**	PC 1	PC 2	PC 3	**Schist**	PC 1	PC 2	PC 3
**Pr**	0.94	0.15	-0.03	**Dy**	0.99	0.09	0.03	**Ce**	0.94	0.04	-0.11	**Y**	0.96	-0.06	-0.07
**Y**	0.90	0.27	0.05	**Tb**	0.98	0.11	0.02	**Pr**	0.92	-0.03	-0.19	**Ho**	0.95	-0.05	-0.05
**Er**	0.89	0.04	-0.02	**Sm**	0.98	0.03	-0.02	**Y**	0.90	0.22	-0.12	**Er**	0.94	-0.08	0.02
**Gd**	0.88	0.07	0.04	**Ho**	0.98	0.10	0.04	**La**	0.88	-0.04	-0.29	**Dy**	0.94	-0.14	-0.04
**Yb**	0.87	-0.19	0.02	**Gd**	0.98	0.04	0.08	**Eu**	0.82	0.21	-0.02	**Pr**	0.92	-0.20	-0.14
**Ce**	0.86	0.30	0.02	**Pr**	0.97	0.00	-0.05	**Gd**	0.78	-0.11	-0.08	**Yb**	0.91	-0.07	0.08
**Dy**	0.85	0.13	0.02	**Yb**	0.97	0.09	0.06	**Dy**	0.67	-0.10	-0.17	**Ce**	0.90	-0.24	-0.13
**La**	0.84	0.29	0.18	**Tm**	0.97	0.06	0.04	**Er**	0.66	-0.21	0.12	**Gd**	0.90	-0.21	-0.09
**Eu**	0.79	-0.22	0.02	**Y**	0.96	0.16	0.09	Ca	0.62	0.26	-0.03	**Sm**	0.89	-0.11	-0.11
**Ho**	0.76	-0.57	-0.04	**Ce**	0.96	-0.10	-0.01	**Yb**	0.61	-0.27	-0.03	**Nd**	0.87	-0.07	-0.03
**Sm**	0.71	-0.40	-0.05	**Lu**	0.96	0.12	0.07	**Sm**	0.56	-0.34	0.04	**La**	0.87	-0.18	-0.15
**Tb**	0.66	-0.65	-0.07	**La**	0.96	0.06	-0.05	**Tb**	0.52	-0.22	0.36	**Tb**	0.87	-0.14	-0.08
HCO_3^-	0.57	0.46	-0.04	**Eu**	0.95	0.08	0.02	**Nd**	0.51	0.10	-0.42	**Lu**	0.75	-0.15	-0.10
Ca	0.44	0.32	0.44	**Er**	0.94	0.11	0.05	**Ho**	0.50	-0.49	0.25	**Eu**	0.72	-0.22	0.02
**Nd**	0.42	0.35	0.17	**Nd**	0.82	0.00	-0.04	Mn	0.44	0.43	0.22	**Tm**	0.64	-0.10	-0.13
**Tm**	0.41	-0.80	-0.05	Fe	0.52	-0.40	-0.48	Al	-0.09	-0.78	-0.11	P	0.63	0.47	0.12
**Lu**	0.53	-0.73	-0.09	Na	0.50	0.48	-0.12	HCO_3^-	0.25	0.67	-0.20	HCO_3^-	0.53	0.76	-0.02
Fe	0.18	0.35	0.26	Si	0.36	-0.30	0.18	**Tm**	0.27	-0.59	0.23	CO_3^{2-}	0.51	0.67	-0.01
P	0.07	0.21	-0.19	TOC	0.20	0.20	0.10	P	0.13	0.58	0.12	Na	0.63	0.64	0.18
H+	-0.13	-0.19	0.76	HCO_3^-	-0.04	0.85	-0.03	H_2CO_3	0.15	0.54	-0.04	Fe	0.28	-0.62	0.36
H_2CO_3	0.30	0.15	0.69	CO_3^{2-}	-0.16	0.78	-0.32	H+	0.04	0.31	0.12	Ca	0.53	0.60	0.47
CO_3^{2-}	0.30	0.42	-0.65	Ca	-0.11	0.72	0.15	Ti	0.14	-0.28	-0.18	Si	0.40	0.50	0.31
Mn	0.13	0.11	0.61	H+	0.19	-0.65	0.59	Si	0.21	0.07	0.77	Ti	0.22	-0.48	0.14
Na	0.45	0.40	-0.60	Al	0.43	-0.65	-0.37	Na	0.31	0.29	0.76	H_2CO_3	0.30	0.41	0.30
Si	0.36	0.20	-0.37	Ti	0.13	-0.45	-0.39	TOC	0.33	0.27	0.56	H+	-0.04	-0.21	0.81
Al	-0.05	0.19	-0.32	H_2CO_3	0.24	-0.22	0.74	**Lu**	0.39	-0.36	0.44	Mn	0.01	-0.10	0.80
TOC	-0.20	-0.21	-0.32	P	0.36	-0.34	-0.41	Fe	0.39	-0.10	-0.41	Al	0.12	-0.54	0.63
Ti	-0.08	0.25	0.29	Mn	-0.18	0.04	0.30	CO_3^{2-}	0.11	0.22	-0.27	TOC	0.18	0.05	0.24

SI 2.3.2. Plant

Table s4. Principal Component Analysis (unrotated) showing relationships between water-normalized REE, trace and major elements in *Buchloe dactyloides* grass roots in different substrates. Correlation values between element and each PC are on a color scale from 1 (red) to 0 (white) and -1 (blue). REE are in bold for easy identification. Elements are in order of their correlation with each component.

Roots														
**Basalt**	PC 1	PC 2	PC 3	**Rhyolite**	PC 1	PC 2	**Granite**	PC 1	PC 2	PC 3	**Schist**	PC 1	PC 2	PC 3
**Tb**	0.94	0.18	0.26	**Lu**	0.99	-0.13	**Y**	0.97	-0.23	0.04	**Sm**	0.99	-0.11	-0.01
**Eu**	0.89	0.22	0.39	**Yb**	0.99	-0.14	**Gd**	0.97	-0.18	0.18	**Dy**	0.98	-0.01	0.15
**Pr**	0.88	-0.45	-0.07	**Er**	0.99	-0.15	**Nd**	0.96	-0.17	-0.09	**Gd**	0.98	-0.02	-0.19
**Er**	0.87	-0.39	0.12	**Y**	0.99	-0.10	**Ce**	0.96	-0.14	-0.09	**Eu**	0.98	0.05	-0.05
**Dy**	0.87	-0.20	-0.35	**Dy**	0.99	-0.16	**La**	0.96	-0.11	-0.23	**Er**	0.97	-0.15	0.06
**Y**	0.82	-0.42	-0.16	**Tm**	0.99	-0.17	**Pr**	0.95	-0.22	-0.10	**Nd**	0.96	0.00	-0.23
**Ce**	0.80	-0.60	0.06	**Ho**	0.99	-0.17	**Ho**	0.95	-0.24	0.20	**Pr**	0.96	0.09	-0.23
**Sr**	0.79	0.57	-0.05	**Tb**	0.98	-0.17	**Dy**	0.94	-0.27	0.21	**Tb**	0.96	-0.23	-0.19
**La**	0.78	-0.42	0.12	**Gd**	0.98	-0.18	**Cu**	0.88	-0.04	-0.38	**Yb**	0.95	-0.13	0.18
**Lu**	0.77	0.12	0.33	P	0.98	-0.15	**Er**	0.86	-0.22	0.42	**Ce**	0.94	0.18	-0.25
**Gd**	0.77	-0.39	0.03	**La**	0.98	-0.21	**Lu**	0.86	-0.40	0.23	**Ho**	0.93	-0.03	-0.19
Na	0.75	0.39	-0.54	**Nd**	0.97	-0.22	**Sm**	0.86	-0.30	0.39	**La**	0.92	0.27	-0.22
Fe	0.74	0.54	0.31	**Sm**	0.97	-0.22	**Eu**	0.81	-0.35	-0.40	**Y**	0.92	0.24	0.25
**Nd**	0.73	0.17	-0.57	**Pr**	0.97	-0.24	**Yb**	0.81	-0.30	0.34	Mn	0.91	0.21	-0.11
**Ho**	0.70	-0.68	-0.01	**Eu**	0.97	-0.21	Al	0.70	0.20	-0.58	**Tm**	0.84	0.31	-0.18
Cr	-0.11	0.89	0.03	Cu	0.97	0.14	**Tm**	0.64	-0.46	0.31	**Lu**	0.80	-0.48	0.05
Si	-0.40	0.88	-0.24	Ti	0.96	-0.22	Fe	0.62	0.53	-0.40	Ca	0.73	0.54	0.17
Ni	0.41	0.83	0.04	**Ce**	0.95	-0.28	**Tb**	0.12	0.99	0.08	Cu	0.73	0.12	0.54
Mg	0.59	0.77	0.00	Si	0.92	0.26	K	0.06	0.92	-0.28	Cr	0.60	0.58	-0.23
**Sm**	0.41	-0.77	-0.31	K	0.90	0.41	Si	0.18	0.92	0.33	Mg	-0.18	0.94	0.27
P	0.23	0.76	-0.38	Zn	0.88	0.40	Ca	0.33	0.91	0.23	Co	0.16	0.89	-0.05
Ca	0.51	0.75	0.07	Na	0.88	0.07	P	0.43	0.88	-0.02	Ti	0.32	-0.85	0.30
Co	0.61	0.73	0.06	Cr	0.86	0.29	Ni	0.48	0.85	-0.12	Fe	0.43	-0.84	0.13
Mn	0.00	-0.69	0.03	Fe	0.85	-0.40	Sr	0.36	0.84	0.39	Na	-0.49	0.77	0.18
**Yb**	0.57	-0.66	0.25	Al	0.77	-0.44	Co	0.49	0.83	-0.19	Al	0.43	-0.76	0.41
K	0.42	0.58	-0.56	Co	0.22	0.97	Ti	0.47	0.71	0.09	P	0.46	-0.74	0.46
Al	0.28	0.57	0.54	Mn	0.26	0.96	Mg	-0.19	-0.10	0.95	Sr	0.57	0.73	0.06
Cu	-0.63	0.19	0.72	Ca	0.55	0.82	Mn	-0.01	0.62	0.77	K	0.48	0.28	0.79
**Tm**	0.31	0.48	0.70	Mg	0.55	0.80	Na	0.27	0.52	-0.72	Si	0.15	0.52	0.58
Zn	-0.25	0.45	-0.58	Sr	0.61	0.78	Cr	-0.10	-0.33	-0.68	Ni	0.47	0.42	-0.09
Ti	0.41	0.32	0.20	Ni	0.65	0.71	Zn	-0.01	-0.17	-0.57	Zn	0.32	-0.56	-0.45

SI **2.4 Plant biomass**

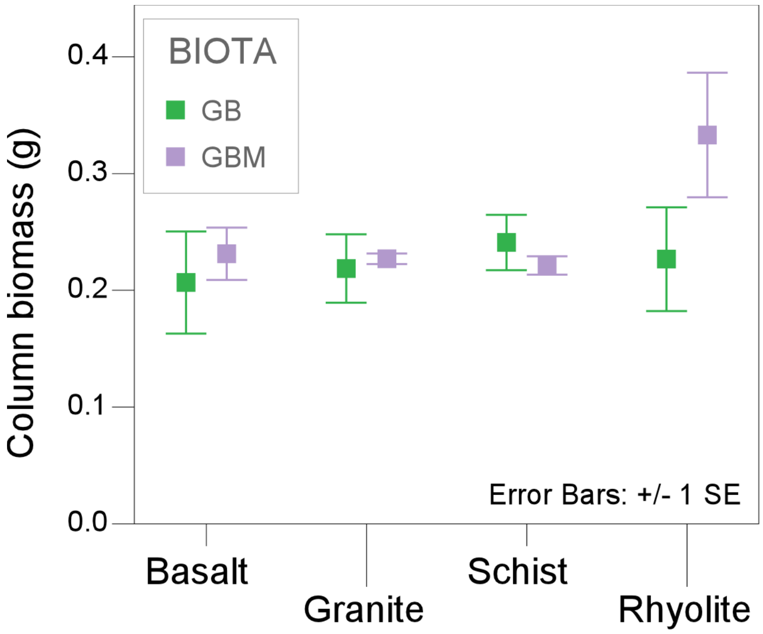

Figure s4. Average column biomass (*Bouteloua dactyloides*) developed on the four rock substrates after the 20-months experiment, as influenced by arbuscular mycorrhiza.

SI **2.5 Coupled denudation, uptake, and stabilization in secondary solid phases**

Table s5. Distribution of REE (sum) in unreacted rock (time=0) and the end of the 20-month experiment for each rock and biotic treatments, together with their ratios mobilized (water + plant) *to* solid phases (AAE + AOE). For each column values were summed across REE series, and for water also across sampling events. Error bars represent +/-1SE. C, abiotic control; B, bacteria; BG, grass-bacteria; BGM, grass-bacteria-mycorrhiza.

% (to rock) weathered REE (mean ±SE)						
		Time 0	C	B	BG	BGM
**Basalt**	Shoot				3.82E-05 ± 8.28E-06	1.11E-04 ± 5.71E-05
	Root				2.72E-04 ± 4.26E-05	3.29E-04 ± 4.81E-05
	Water		2.38E-05 ± 4.90E-07	3.48E-05 ± 5.44E-06	5.24E-05 ± 1.20E-05	6.20E-05 ± 3.07E-06
	AAE	0.271 ± 0.003	0.233 ± 0.004	0.229 ± 0.005	0.231 ± 0.002	0.227 ± 0.002
	AOE	0.297 ± 0.005	0.505 ± 0.034	0.537 ± 0.018	0.499 ± 0.008	0.624 ± 0.059
	Unextracted residual	99.43 ± 0.66	99.26 ± 0.0	99.23 ± 0.0	99.27 ± 0.0	99.15 ± 0.1
**Rhyolite**	Shoot				3.86E-04 ± 1.22E-04	5.24E-04 ± 8.79E-05
	Root				2.79E-03 ± 4.90E-04	4.50E-03 ± 1.32E-03
	Water		5.32E-04 ± 6.23E-05	1.78E-03 ± 2.58E-04	2.47E-03 ± 3.69E-04	1.16E-03 ± 1.25E-04
	AAE	1.39 ± 0.03	1.16 ± 0.02	0.845 ± 0.018	0.835 ± 0.056	1.06 ± 0.04
	AOE	9.76 ± 0.15	5.96 ± 0.06	4.69 ± 0.11	5.81 ± 0.10	6.08 ± 0.04
	Unextracted residual	88.85 ± 1.88	92.88 ± 0.0	94.46 ± 0.1	93.34 ± 0.2	92.85 ± 0.1
**Granite**	Shoot				1.24E-04 ± 3.98E-05	6.95E-05 ± 2.31E-05
	Root				1.67E-04 ± 3.28E-05	2.80E-04 ± 5.74E-05
	Water		4.85E-05 ± 2.29E-06	5.20E-05 ± 3.66E-06	1.47E-04 ± 4.19E-05	1.30E-04 ± 2.73E-05
	AAE	0.26 ± 0.01	0.0665 ± 0.0010	0.0675 ± 0.0013	0.0579 ± 0.0016	0.0666 ± 0.0025
	AOE	0.20 ± 0.00	0.0616 ± 0.0042	0.0700 ± 0.0087	0.0658 ± 0.0018	0.0653 ± 0.0031
	Unextracted residual	99.55 ± 2.49	99.87 ± 0.0	99.86 ± 0.0	99.88 ± 0.0	99.87 ± 0.0
**Schist**	Shoot				2.00E-04 ± 7.11E-06	2.84E-04 ± 5.45E-05
	Root				1.88E-03 ± 2.76E-04	2.20E-03 ± 2.87E-04
	Water		2.09E-04 ± 2.36E-05	1.74E-04 ± 1.29E-05	2.08E-04 ± 2.68E-05	2.59E-04 ± 5.39E-05
	AAE	0.12 ± 0.00	0.0432 ± 0.0010	0.0373 ± 0.0015	0.0748 ± 0.0033	0.0531 ± 0.0090
	AOE	0.44 ± 0.02	0.160 ± 0.005	0.168 ± 0.005	0.227 ± 0.010	0.201 ± 0.012
	Unextracted residual	99.45 ± 2.72	99.80 ± 0.0	99.79 ± 0.0	99.70 ± 0.0	99.74 ± 0.0
Basalt	Mobilized / solid		0.000032	0.000046	0.000496	0.000590
Rhyolite	Mobilized / solid		0.000075	0.000321	0.000849	0.000864
Granite	Mobilized / solid		0.000378	0.000378	0.003532	0.003638
Schist	Mobilized / solid		0.001031	0.000846	0.007560	0.010809

N (for each average) = 3 columns

SI References

1. Zaharescu, D. G. Modular environmental chamber. United States Patent and Trademark Office, US 61/982,318 (2016).
2. Burghelea, C. I. *et al.* Mineral nutrient mobilization by plant from rock: Influence of rock type and arbuscular mycorrhiza. *Biogeochemistry* **124,** 187–203 (2015).
3. Gaillardet, J., Duprre, B., Louvat, P. & Allegre, C. J. Global silicate weathering and CO2 consumption rates deduced from the chemistry of large rivers. *Chem. Geol.* **159,** 3–30 (1999).
4. Suchet, P. A. Worldwide distribution of continental rock lithology: Implications for the atmospheric/soil CO 2 uptake by continental weathering and alkalinity river transport to the oceans. *Global Biogeochem. Cycles* **17,** (2003).
5. Rudnick, R. & Gao, S. C omposition of the Continental Crust. *Treatise on Geochemistry* **3,** 1–64 (2003).
